# Evaluation of the effects of subgingival injection of Simvastatin on space re-opening after orthodontic space closure in adults

**DOI:** 10.15171/joddd.2016.001

**Published:** 2016-03-16

**Authors:** Arezoo Jahanbin, Mostafa Abtahi, Parastoo Namdar, Farzin Heravi, Fatemeh Sadeghi, Hamidreza Arab, Hooman Shafaee

**Affiliations:** ^1^Associate Professor, Dental Research Center, Department of Orthodontics, School of Dentistry, Mashhad University of Medical Sciences, Mashhad, Iran; ^2^Associate Professor, Dental Material Research Center, Department of Orthodontics, School of Dentistry, Mashhad University of Medical Sciences, Mashhad, Iran; ^3^Assistant Professor, Department of Orthodontics, School of Dentistry, Mazandaran University of Medical Sciences, Sari, Iran; ^4^Professor, Dental Research Center, Department of Orthodontics, School of Dentistry, Mashhad University of Medical Sciences, Mashhad, Iran; ^5^Professor,Department of Pharmaceutics, Pharmacology School, Mashhad University of Medical Sciences, Mashhad, Iran; ^6^Associate Professor, Dental Research Center, Department of Periodontics, School of Dentistry, Mashhad University of Medical Sciences, Mashhad, Iran; ^7^Assistant Professor, Oral & Maxillofacial Diseases Research Center, Department of Orthodontics, School of Dentistry, Mashhad University of Medical Sciences, Mashhad, Iran

**Keywords:** Periodontal index, relapse, statins, tooth movement

## Abstract

***Background.*** This clinical trial evaluated the effect of Simvastatin on space re-opening after orthodontic space closure and its effect on the gingival index (GI) and clinical attachment loss (CAL).

***Methods.*** 16 females, 25-40 years old, with spaces between anterior mandibular teeth due to chronic periodontitis were participated in this study. The patients were randomly divided into control and experimental groups. In the experimental group, 1.2% Simvastatin gel and in the control group, 0.9% sodium chloride as a placebo was injected into the pocket depth of the six anterior teeth. The amount of space reopening, GI and CAL were measured.

***Results.*** No serious complications were observed during interventions and follow-up periods. Space re-opening was significantly reduced in patients receiving Simvastatin (P < 0.001). Moreover, GI reduction was significantly greater in Sim-vastatin group compared to the control group (P < 0.001). However, CAL did not demonstrate a significant difference between the groups.

***Conclusion.*** Simvastatin may decrease space re-opening after orthodontic space closure in human anterior teeth.

## Introduction

There is a great tendency to relapse after orthodontic tooth movement.^1^ Contemporary retaining strategies in orthodontics basically include removable and fixed retainers. Removable retainers facilitate oral hygiene; however, their most important drawback is patient compliance. On the other hand, fixed retainers, which are usually used for long-term retention after orthodontic treatment, make oral hygiene more difficult.^2^

Considering these problems, a few recent studies have suggested that pharmacologic therapy might provide another mechanism to control orthodontic relapse.^3-5^ One of these drugs is Simvastatin (SMV), which is widely used for lowering serum cholesterol.^6^ SMV has an anabolic effect in vivo.^7^ Also, it seems to promote bone production by enhancing the expression of bone morphogenic protein-2 and angiogenesis.^7,8^ Various investigation on animals have reported that applying SMV locally had the potential to stimulate bone regeneration and an anti-inflammatory effect.^9-11^ Furthermore, an in vivo study reported that SMV helps in bone formation in the alveolus of rats with periodontitis.^12^Han et al^13^showed that the amount of orthodontic relapse was decreased in the rats treated by this drug compared with the controls. Therefore, SMV may provide a new direction in controlling relapse of orthodontic treated cases.

The aim of this clinical trial was to examine the effect of Simvastatin on space re-opening after orthodontic space closure and its effect on gingival index (GI) and clinical attachment level (CAL).

## Methods

This study was accepted by the Ethics Committee of the Mashhad University of Medical Sciences (No 900303). This was a parallel-group, double blind, single-center, randomized controlled clinical trial, with a 1:1 allocation ratio. Female patients, between 20 to 45 years of age, who referred to the Department of Periodontics at Mashhad School of Dentistry were selected. Patients’ recruitment commenced in November 2011 and ended in May 2012. Patients with controlled chronic moderate periodontitis (4<CAL ≤5) and diffuse spacing (4-6mm) between their anterior teeth (from mesial of left canine to mesial of right canine) in lower arch were included. Exclusion criteria were systemic use of Statins, any systemic disease, severe periodontitis (CAL > 5 mm), spacing with etiology other than periodontitis, rotation of the anterior teeth, crown to root ratio greater than 1:1, pregnant or lactating women and allergy to Statins.

After primary case selection, a detailed document including demographic data and patient medical and dental history was filled out and each patient signed an informed consent form.

Subsequently, the spaces within six mandibular anterior teeth were measured by a caliper (Dentaurum, Inspringen, Germany) with 0.01 mm accuracy and at last twenty six female patients (mean age, 39 years; range 20-45 years) were entered into the study and randomized in a 1:1 allocation ratio to either experimental or control group.

A standard occlusal photograph was taken with a digital camera (Canon Powershot A540). An alginate impression (HeraeusKulzer Ltd, Bayer, Germany) was taken from the mandibular arches and impressions were poured with Velmix stone (Vel-Mix-Pink Die Stone, Kerr Dental laboratory, CA, USA) in order to make a study model.

CAL and the GI were recorded using a periodontal probe by a periodontist. CAL was calculated by measuring the pocket depth plus the distance of the CEJ to free gingiva. In this study the mesiobuccal, midbuccal, distobuccal, mesiolingual, midlingual, and distolingual points of each anterior teeth were probed and GI was recorded.^14^

Two weeks before the placement of orthodontic attachments, scaling and root planning was performed for all of the patients.

Bonded tubes (Dentaurum, Inspringen, Germany) were bonded on the buccal surface of the first molars, and brackets (Roth prescription 0.018"; Dentaurum, Inspringen, Germany) were bonded on the other teeth. After initial alignment by NiTi wires (Dentaurum, Inspringen, Germany), two stops were inserted in front of the molar tubes to prevent the decrease in arch length. Anterior spaces were closed using an elastic chain (Dentaurum, Inspringen, Germany) from the right to left canine on 0.016 inch SS base archwire (Dentaurum, Inspringen, Germany). Therefore, anterior spaces were closed without decrease in arch length and spaces gathered distal to the right and left canines. The purposes of space closure were esthetic and function because the spaced teeth were migrated labially and were prone to bone loss.

In both groups, mandibular anterior teeth (canine to canine) were ligated with ligature wire for four weeks and a second impression and occlusal photograph was taken and CAL and GI was recorded again. In this stage, one patient from control group refused to continue treatment and was removed from the study. In the experimental group, consisting 13 patients, 0.1 ml of 1.2% SMV gel was injected into the pocket of all six mandibular anterior teeth using an opaque insulin syringe. SMV gel was prepared based on the study of Pradeep&Throat.^15^

In the control group, consisting of 12 dental arches, 0.1 ml NaCl (0.9%) as a placebo was injected into the pocket depth of the mandibular anterior teeth. In both groups, four weeks after injection, the ligature and the base wire were removed for the duration of four weeks. Study models were made and occlusal-view photographs were taken eight weeks after the injection and CAL and GI were recorded again at this time. For measuring anterior space re-opening four weeks after the removal of wires, the occlusal photographs were analyzed to measure the variables on them using Smile Analyzer software. Thissoftwarehasa capability forexactmeasurementofdesireddistancesor anglesonimagesandradiographs.^16^

The amount of re-opened spaces was measured and for determining magnification, the width of one incisor tooth from the study cast was used. For assurance, the spaces were measured again with a caliper on dental stones and there was no significant difference between the two methods (P = 0.49). To determine inter-examiner reliability in measuring spaces and recording GI and CAL, five subjects were randomly selected and examined by a second observer. To establish intra-examiner reliability, another five subjects were randomly selected and reevaluated by the first observer. Strong inter-examiner (kappa coefficient = 0.88, P < 0.001) and intra-examiner (kappa coefficient = 0.92, P < 0.001) agreements were observed on all variables.

Finally, all spaces were closed using elastic chain from right to left molar and a canine-to-canine fiber reinforced composite(FRC) fixed retainer (Angelus, Londrina, Brazil) was bonded.

Because of difference in texture of SMV gel and NaCl, injections were delivered in an opaque syringe. Therefore, both the operator and the patient were blinded to the study. Also, blinding was applied for outcomes evaluation.

Data were analyzed by SPSS software (version 16.0, Chicago, USA).To control the normality of data, Kolmogrov-Smirnov test was used. The independent t-test and Chi-square tests were used at a significance level of 0.05.

## Results

Before treatment, initial spaces between the mandibular six anterior teeth of the patients were not statistically significant different between two groups (P=0.330). Also, the mean age of cases in the experimental and control groups showed no statistically significant difference (P =0.45). GI and CAL also showed no significant differences before the beginning of orthodontic treatment between the two groups (P = 0.51 and P = 0.63, respectively).

Four weeks after removal of the ligature and base wire, mandibular anterior spaces were measured in each group. In the control group, the re-opened spaces were nearly 5.5 times more in the experimental group (P < 0.001; Table 1).

**Table 1:** Mean (SD) re-opened spaces eight weeks after injection in both groups

**Groups**	**Number of reopened cases**	**Mean (SD) reopened space (mm)**	**Statistical significance**
**Experimental group (N = 13)**	7	0.29(0.32)	P < 0.001; T=4.8
**Control group (N =12)**	12	1.73(1)

^*^Sum of re-opened spaces

There was no relationship between the initial spaces less than 5 mm and the amount of space opening. A positive relationship existed between the initial spaces greater than 5 mm and the amount of space re-opening. When initial spaces were greater than 5 mm, the amount of space re-opening was greatly increased. In the SMV group in 53%, and in the control group, in 100% of cases, spaces re-opened after treatment and the difference was statistically significant (P = 0.015).

After closing the spaces in both groups, GI was not significantly different (P = 0.43), but eight weeks after receiving an SMV gel injection, GI in the SMV group was significantly reduced (P < 0.001). At this time, the depletion in GI was significantly greater in the experimental group in comparison to the control group (P < 0.001; Table 2).

**Table 2:** Mean (SD) gingival index (GI) before and eight weeks after injection in both groups

**Groups**	**Before**	**After**	**Reduction in GI**
**Experimental group (N = 13)**	1.62 ± 0.45	1.04 ± 0.43	0.5808 ± 0.333
**Control group (N = 12)**	1.75 ± 0.32	1.73 ± 0.31	0.0167 ± 0.046
**Statistical significance**	P = 0.43; t=0.79	P < 0.001; t=4.5	P < 0.001; t=6.03

No significant difference was found in CAL between the two groups before the injection and eight weeks after the injection with SMV gel and sodium chloride (P > 0.05; Table 3).

**Table 3:** Mean (SD) clinical attachment loss (CAL; mm) and reduction in gingival index (GI) before and eight weeks after injection

**Groups**	**CAL before**	**CAL after**	**Reduction in GI**
**Experimental group (N = 13)**	4.07 ± 0.52	3.84 ± 0.46	0.23 ± 0.18
**Control group (N = 12)**	4.19 ± 0.34	4.07 ± 0.39	0.11 ± 0.28
**Statistical Significance**	t = 0.66; P=0.51	t = 1.36; P=0.18	t = 1.24; P=0.22

## Discussion

Retention is one of the most challenging parts in orthodontics. Moreover, retention, which is the procedure by which orthodontists try to minimize relapse following treatment, is an important phase of orthodontic treatment.^17^

Many factors such as periodontal, occlusal, tissue pressure and growth are related to the etiology of relapse.^18^Even though the trans-septal fibers are originally responsible for the production of forces, osteoblastic and osteoclastic bone activity is essential for relapse. Therefore, provocation of alveolar bone production or restriction of bone resorption after orthodontic tooth movement may prevent relapse. To shorten the retention period, pharmacologic methods, which affect bone remodeling, were introduced.^13^

In our study, we designed a randomized clinical trial to investigate the effect of SMV on short-term relapse in patients with diffuse spacing. Our findings showed, eight weeks after injection of SMV, the amount of relapse was significantly lesser in the experimental than the control group. This finding indicated that SMV could reduce relapse tendency. In this study, relapse was examined four weeks post orthodontic treatment because previous studies have shown that the rate of relapse is faster in the first week.^13,19^

In addition, many studies have showed that SMV promoted bone formation and inhibited osteoclastic activity.^20,21^ Mundy et al^7^ first demonstrated that Statins could stimulate new bone regeneration in rodents and it is related to an increased expression level of bone morphogenic protein-2 in osteogenic cells. Han et al^13^ reported that SMV prevents osteoclasts while stimulating bone formation, probably by regulating the ratio of local osteoprotegerin to RANKL in the periodontal tissue. They concluded that SMV might be useful for retention.^13^ According to von Knoch et al,^22^both bisphosphonates and Statins are effective anti-resorptive agents. The concentration of SMV used in our study was 1.2 mg, based on the study of Pradeep & Thorat.^15^Yazawaet al^23^reported that SMV in low concentrations exhibited a positive effect on reproduction and differentiation of PDL cells to osteoblasts. Mirhashemiet al^24^ evaluated the impact of Atrovastatin on orthodontic tooth movement in male Wistarrat and reported that Atrovastatin appears to reduce tooth movement in rats. This result was in agreement with our study, in which the space re-opening was reduced after injection of Simvastatin.

In the experimental group of our study, GI was reduced significantly in comparison to the control group, which probably relate to the anti-inflammatory effect of SMV.^15,25^ Lindy et al^26^ reported that pathologic periodontal lesions in patients treated by systemic statins were 37% less than in the control group. Sokodaet al^10^ showed that SMV reduced the generation of IL-6 and IL-8, indicating an anti-inflammatory feature for it. Pradeep & Thorat^15^showed that there was a higher decrease in the GI and probing depth, and more CAL gain with significant intrabony defect improvements at places treated with scaling root planning plus locally injection of SMV in patients suffering chronic periodontitis. However, in our study eight weeks after an injection of SMV, there was no significant reduction in CAL.

As we mentioned, our samples were selected from patients with controlled moderate periodontitis because these patients most often have spaces between their incisors and their chief complaint is anterior spacing. Moreover, a study showed that a subgingival injection of SMV may reduce CAL and the GI in such patients.^15^ Besides, FRC retainers used after space closure may be beneficial for these patients because they act as a splint.

Long-term studies using different concentrations of SMV should be performed to assert the observations of our study. We suggest evaluating the effect of SMV in anchorage reinforcement in the future.

## Conclusion

Subgingival injection of SMV during orthodontic tooth movement could decrease space re-opening and GI, but may have no effect on CAL. The results indicated the capability of SMV in reducing relapse after anterior space closure.

## Acknowledgments

This paper was written based on a MSc undergraduate student thesis registered at Mashhad University of Medical Sciences (thesis no. 506). The authors would like to acknowledge the Vice-Chancellor for Research at Mashhad University of Medical Sciences for the financial support of this project.

## Authors’ contributions

AJ was responsible for the concept and study design. MA performed study experiments. PN also performed study experiments and the analysis. FH performed the literature review and revised the manuscript. FS provided pharmacologic consult. HS was responsible for drafting the manuscript. HA critically revised the manuscript. All authors have read and approved the final manuscript.

## Funding

The Vice-Chancellor for Research at Mashhad University of Medical Sciences supported this project.

## Competing interests

The authors declare that they have no competing interests with regards to the authorship and/or publication of this article.

## Ethics approval

This study was approved by the ethics committee of the Mashhad University of Medical Sciences (No 900303).
